# Role of Endolysosomes in Skeletal Muscle Pathology Observed in a Cholesterol-Fed Rabbit Model of Alzheimer’s Disease

**DOI:** 10.3389/fnagi.2016.00129

**Published:** 2016-06-08

**Authors:** Xuesong Chen, John F. Wagener, Othman Ghribi, Jonathan D. Geiger

**Affiliations:** Department of Basic Biomedical Sciences, School of Medicine and Health Sciences, University of North DakotaGrand Forks, ND, USA

**Keywords:** Alzheimer’s disease, muscle, LDL, endolysosome, amyloid beta, phosphorylated tau, ubiquitin

## Abstract

Deficits in skeletal muscles contribute not only to the functional decline in people living with Alzheimer’s disease (AD), but also to AD pathogenesis. We have shown that endolysosome dysfunction plays an important role in the development of AD pathological features in a cholesterol-fed rabbit model of AD. Interestingly we observed in skeletal muscle from the rabbit AD model increased deposition of Aβ, phosphorylated tau, and ubiquitin. Here, we tested the hypothesis that endolysosome dysfunction commonly occurs in skeletal muscle and brain in this rabbit model of AD. In skeletal muscle of rabbits fed a 2% cholesterol-enriched diet for 12 weeks we observed the presence of abnormally enlarged endolysosomes, in which were increased accumulations of free cholesterol and multiple AD marker proteins subject to misfolding and aggregation including Aβ, phosphorylated tau, and ubiquitin. Moreover, in skeletal muscle of rabbits fed the cholesterol-enriched diet we observed decreased specific activities of three different lysosome enzymes. Our results suggest that elevated levels of plasma cholesterol can disturb endolysosome structure and function as well as promote the development of AD-like pathological features in skeletal muscle and that these organellar changes might contribute to the development of skeletal muscle deficits in AD.

## Introduction

Alzheimer’s disease (AD) is the most common neurodegenerative disorder of old age that results in massive health care costs in the United States (Hebert et al., 2013). AD is characterized clinically by a progressive cognitive impairment and pathologically by neurodegeneration and the presence of amyloid plaques composed of amyloid beta (Aβ) protein and neurofibrillary tangles composed of phosphorylated tau (Holtzman et al., 2011; Goate and Hardy, 2012). Besides neurodegeneration and cognitive impairment, AD patients often exhibit loss of muscle mass and reduced muscle strength, and such deficits in skeletal muscle may be early signs of AD and as such these deficits may help predict the onset and progression of clinical AD (Buchman et al., 2007; Boyle et al., 2009; Burns et al., 2010). Furthermore, skeletal muscle dysfunction can lead to progressive functional problems in AD patients and some have proposed that these changes may be not only concordant to those occurring in brain, but also that the skeletal muscle changes may serve as a potential biomarker for the onset and progression of AD (4–6). Although it is not known how such skeletal muscle deficits are developed in AD, a testable hypothesis is that common pathogenic processes occur in brain as well as skeletal muscle and that these changes may help explain observations of elevated levels of Aβ in skeletal muscle of AD patients (Kuo et al., 2000).

Altered cholesterol homeostasis in general and elevated plasma LDL cholesterol in specific continues to represent robust risk factors of sporadic AD (Solomon et al., 2009; Chen et al., 2010; Lesser et al., 2011; Hui et al., 2012; Reed et al., 2014). Others and we have shown that rabbits fed a cholesterol-enriched diet exhibit pathological hallmarks of AD including increased levels of Aβ, phosphorylated tau, and synaptic disruption (Sparks et al., 1994; Ghribi et al., 2006; Chen et al., 2010). Mechanistically, we demonstrated in such rabbits that elevated levels of plasma cholesterol disrupted blood–brain barrier integrity (Ghribi, 2006; Chen et al., 2008a), increased brain levels and the neuronal endolysosome accumulation of apoB (the exclusive apolipoprotein of LDL cholesterol that is normally found only in the periphery), disturbed the structure and function of neuronal endolysosomes, and led to the appearance of pathological features of AD including disrupted synaptic integrity, brain deposition of Aβ, and tau pathology (Chen et al., 2010). Thus, LDL cholesterol coming from the systemic circulation might be altering neuronal endolysosome function and contributing to the pathogenesis of AD. Furthermore, using primary cultured neurons, we demonstrated that LDL cholesterol treatment increased endolysosome accumulation of cholesterol, enlarged the sizes and numbers of endolysosomes, and elevated endolysosome pH (Hui et al., 2012). Moreover, we demonstrated that such alterations in the structure and function of endolysosomes were directly involved in Aβ deposition, tau pathology, and disrupted synaptic integrity (Hui et al., 2012). Thus, our findings suggest strongly that elevated levels of LDL cholesterol contribute to pathogenesis of AD by disturbing the structure and function of neuronal endolysosomes.

Cholesterol for neurons is supplied by astrocyte-derived lipoproteins. Similarly, cholesterol for skeletal muscle is supplied by plasma lipoproteins (Spady and Dietschy, 1983). Extracellular cholesterol, mainly in the form of LDL particles, is delivered into muscle cells via receptor-mediated endocytosis and transported to endolysosomes (Brown and Goldstein, 1986). Importantly, we have shown that the same cholesterol-fed rabbits that develop AD-like pathology in brain (Chen et al., 2010) exhibit increased deposition of Aβ and phosphorylated tau in skeletal muscle fibers (Chen et al., 2008b). Because of the link between LDL cholesterol and endolysosomes and because altered endolysosome dysfunction plays an important and early role in the pathogenesis of AD (Nixon, 2005; Rajendran et al., 2008; Shimizu et al., 2008; Sannerud et al., 2011; Wolfe et al., 2013), we hypothesize that elevated levels of plasma LDL cholesterol disturb the structure and function of endolysosomes and promotes to the development of AD-like pathological features in skeletal muscle thus contributing to the skeletal muscle deficit observed in AD. Herein, we report that cholesterol-enriched diet induced morphological and functional changes of endolysosomes as well as abnormal accumulations of ubiquitin, phosphorylated tau, and Aβ/AβPP in skeletal muscle endolysosomes.

## Materials and Methods

### Rabbits

New Zealand white female rabbits (1.5 to 2 years old) weighing 3 to 4 kg were fed either normal chow (*n* = 5) or a normal chow supplemented with 2% cholesterol (*n* = 9). After 12 weeks on the diet, animals were anesthetized and euthanized, and skeletal muscle (triceps) was dissected, frozen on liquid nitrogen cooled surface, and stored at -80°C until taken for experimentation. The animal protocol was approved by the University of North Dakota Animal Care and Use Committee adherent with the Guide for the Care and Use of Laboratory Animals (NIH publication number 80–23).

### Immunohistochemistry

Cryostat-sectioned skeletal muscle (thickness 14 μm) was stained for target proteins using antibodies to EEA1 (Santa Cruz, sc-0415), LAMP2 (Santa Cruz, sc-8101), cathepsin D (Sigma, c0715), Aβ (4G8, Signet, 39220), phosphorylated tau (SMI-31, Covance, 141815001), ubiquitin (Santa Cruz, sc-8017), N-terminal AβPP (Chemicon, MAB348), C-terminal AβPP (Sigma, A8717), and microtubule-associated protein light chain 3 (LC3, Santa cruz, sc-16755). For immunohistochemistry for bright field microscopy, sections were developed with diaminobenzidine substrate using the avidin-biotin horseradish peroxidase system (Vector Laboratories) and counterstained with hematoxylin, and bright field images were taken by a Nikon Eclipse 80i (upright) microscope with a 40× PlanApo objective. Double fluorescence staining was used to determine co-localization of early endosome marker (EEA1, Abcam ab70521) with late endosome/lysosome marker (LAMP2, Santa Cruz, sc-8101) or autophagosome marker (LC3, Santa cruz, sc-16755) and subcellular co-distribution patterns of AβPP, Aβ, phosphorylated tau, and ubiquitin in early endosome EEA1 (Santa Cruz, sc-0415) and lysosomes (LAMP2, Santa Cruz, sc-8101) or autophagosomes (LC3, Santa cruz, sc-16755). Sections were examined by an Olympus FV300 laser scanning confocal microscope: Argon laser (488 nm, 10 mW) and HeNe laser (543 nm, 1 mW), external two-channel photomultiplier detection, FITC and Texas red probes, 60× oil PlanApo objective, Spot RT color CCD camera, and Fluoview software. Free cholesterol was stained with filipin (Sigma) and co-distribution of free cholesterol with endolysosomes was examined with a Leica DM4000B fluorescent microscope: 63× oil HCX PL Fluotar objective, DAPI and Texas red filters, Leica SCR camera, Leica Application Suite software. Images were process by Image J software or Photoshop CS5 software. Controls for specificity were used including staining muscle with an isotype-matched irrelevant antibody as a negative control, staining muscle with primary antibodies without fluorescence-conjugated secondary antibodies (background controls), and staining muscle with only secondary antibodies – these controls allowed us to eliminate auto-fluorescence in each channel and bleed-through (crossover) between channels.

### Immunoblotting

Skeletal muscle was homogenized mechanically in TPER extraction buffer (Pierce) at a ratio of 1:20 (w:v) in the presence of protease inhibitor cocktail (Sigma) and phosphatase inhibitors (5 mmol/l sodium fluoride and 50 μmol/l sodium orthovanadate). The detergent-soluble fraction (supernatant) was isolated by centrifugation at 100,000 × g for 1 h at 4°C. Protein concentration was determined by Bradford assay. Equal amounts of protein (100 μg) from detergent-soluble fractions were resolved by SDS-PAGE under reducing conditions, transferred to PVDF membranes, and subjected to immunoblotting with antibodies to N-terminal AβPP (1:1000, Chemicon, MAB348), phosphorylated tau (AT8, 1: 1000, Pierce, MN1020), tau 5 (1:1000, Calbiochem 577801), acid phosphatase (1:1000, Abcam, ab54720), cathepsin D (1:1000, Sigma, C0715), cathepsin B (1:1000, Sigma, C6243), Aβ (6E10, 1:500, Signet, 9320), and LC3 was detected using an anti-LC3 antibody (which recognizes both LC3-I and LC3-II, 1:500, Abcam, ab58610). Blots were probed with secondary antibodies conjugated with horseradish peroxidase (HRP) for 1 h at room temperature, reacted with luminal reagent, exposed, visualized, and analyzed by LabWorks 4.5 software on a UVP Bioimaging System (Upland). Quantification was performed by densitometry and the results were analyzed and normalized (vs. averaged densitometric volume values of control rabbits). Glyceraldehyde 3-phosphate dehydrogenase (GAPDH, 1:5000, Abcam, ab8245) was used for loading controls.

### Lysosomal Enzyme Activity Measurement

Acid phosphatase enzyme activity was determined using an Acid Phosphatase Assay kit (Sigma); a luminescence-based assay that uses 4-nitrophenyl phosphate as the substrate (Chen et al., 2010). Enzyme activities of cathepsin D and cathepsin B were determined using two separate assay kits (BioVision); fluorescence-based assays that use preferred MCA-labeled substrates for cathepsin-D and cathepsin B (Chen et al., 2010). Enzyme activities were expressed as relative opitcal density or fluorescence units (RFU) per 50 μg of total protein. Specific activities of each enzyme were indicated by the ratio of enzyme activity to protein levels as determined by immunoblotting

### Statistical Analysis

All data were expressed as mean and SEM. Statistical significance was determined with unpaired two-tail Student’s *t*-test (Frequentist tests indicated that all data were normally distributed). *P* < 0.05 was considered to be statistically significant.

## Results

### Cholesterol-Enriched Diet Increases Accumulation of Cholesterol in Endolysosomes and Disturbs Endolysosome Structure and Function

**FIGURE 1:**
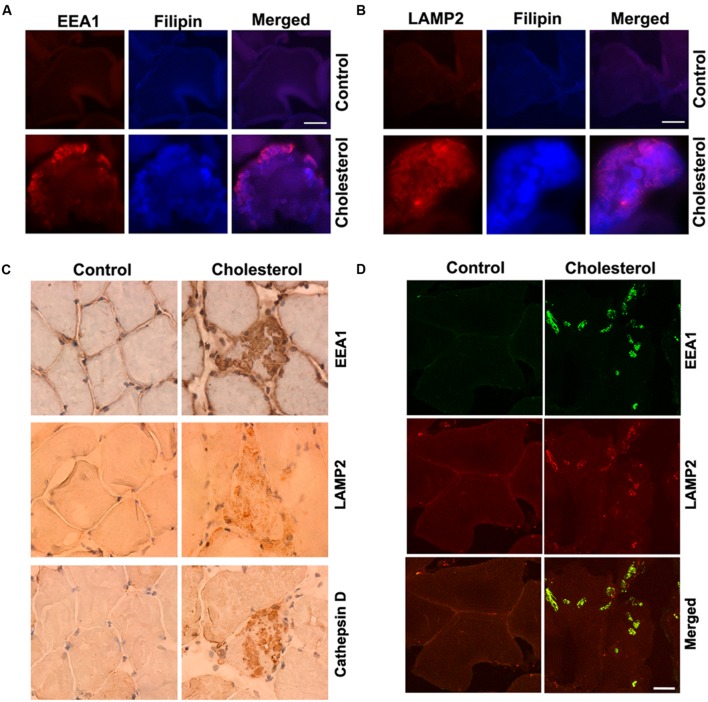
**Cholesterol-enriched diet increases accumulation of free cholesterol in endolysosomes and induces endolysosome enlargement. (A,B)** Filipin-positive staining of free cholesterol (blue) co-distributed with EEA1-positive endosomes (red) and LAMP2-positive lysosomes (red) in muscle from cholesterol-fed rabbits. **(C)** In muscle fibers from control rabbits, EEA1, LAMP2, and cathepsin D staining appeared weak and diffuse, but in muscle fibers from cholesterol-fed rabbits, EEA1, LAMP2, and cathepsin D positive staining was strong forming large clumps (40X). **(D)** Cholesterol-enriched diet increased co-localization of EEA1 with LAMP2 in those abnormally enlarged endolysosomes. Bar = 20 μm.

Skeletal muscle has a very low capacity to synthesize cholesterol (Spady and Dietschy, 1983) and it meets its cholesterol needs mainly by taking up circulating lipoproteins. Extracellular cholesterol in the form of LDL is delivered into muscle cells via receptor-mediated endocytosis and transported to endolysosomes (Brown and Goldstein, 1986). In our cholesterol-fed rabbits, serum levels of cholesterol were about 10-times higher than normal (Chen et al., 2008b). Because LDL cholesterol is endocytosed in skeletal muscle, we examined the extent to which cholesterol was accumulated in endolysosomes. Using double fluorescence staining techniques with EEA1 as a marker for endosomes, filipin for free cholesterol, and LAMP2 for lysosomes, we found that filipin-positive staining of free cholesterol co-distributed with EEA1-positive endosomes (**Figure 1A**) and LAMP2-positive lysosomes (**Figure 1B**). None of these features were present in muscle from control rabbits. In addition, we found that EEA1-positive endosomes and LAMP2-positive lysosomes appeared to be enlarged (**Figures 1A,B**). To access further endolysosome morphological changes we stained for EEA1, LAMP2, and the lysosomal enzyme cathepsin D and found under light microscopy that endolysosome-positive signals (EEA1, LAMP2, and cathepsin D) were weak and diffuse in muscle from control rabbits, but endolysosome-positive staining was readily observed and appeared as large specifically stained aggregates in muscles from cholesterol-fed rabbits (**Figure 1C**). Furthermore, we found that the percentage of muscle fibers that contained such abnormally enlarged endolysosomes was 2.5 ± 0.6% (% to total muscle fibers examined). The percentage of impaired muscle fibers seems low, however, it is induced by cholesterol diet treatment for only 3 months. With longer treatment, we expect more muscle fibers will be affected, and this could have a significant impact for the pathogenesis of sporadic AD, which take decades to develop.

It is not clear how such abnormally enlarged endolysosomes are developed; however, based on recent findings that EEA1 positive early endosomes could fuse with lysosomes under disrupted endocytic homeostasis (Falcon-Perez et al., 2005; Das and Pellett, 2011; Ramanathan et al., 2013), the observed abnormally enlarged endolysosomes in cholesterol fed rabbits could result from uncontrolled fusion of endosomes with late endolysosome/lysosomes. As such, we determined the co-localization of EEA1 with LAMP2, and we found that EEA1 is co-localized with LAMP2 in those abnormally enlarged endolysosomes in muscle fibers from cholesterol-fed rabbits (**Figure 1D**). These findings suggest that increased cholesterol uptake and subsequent endolysosome accumulation of cholesterol in skeletal muscle could disrupt endocytic homeostasis and lead to uncontrolled fusion of early endosomes with late endosomes/lysosomes.

**FIGURE 2:**
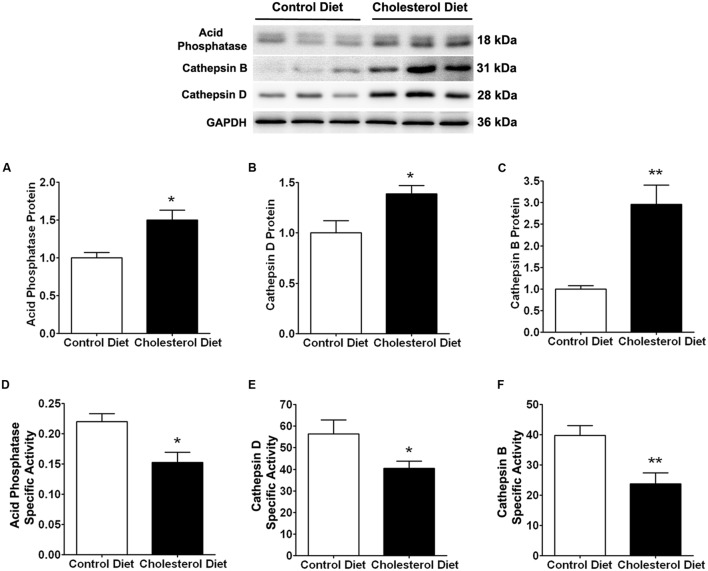
**Cholesterol-enriched diet impairs specific activities of endolysosome enzymes**. Representative western blots were shown. The cholesterol-enriched diet (*n* = 9) increased significantly protein levels of acid phosphatase (**A**, ^∗^*p* < 0.05), cathepsin D (**C**, ^∗^*p* < 0.05), and cathepsin B (**E**, ^∗∗^*p* < 0.01) in skeletal muscle, when compared with controls (*n* = 5). Cholesterol-enriched diet (*n* = 9) decreased significantly specific activities (ratio of total enzyme activity to protein levels as determined by immunoblotting) of acid phosphatase (**B**, ^∗^*p* < 0.05), cathepsin D (**D**, ^∗^*p* < 0.05), and cathepsin B (**F**, ^∗∗^*p* < 0.01), when compared with controls (*n* = 5).

Because of the above findings that cholesterol-enriched diet induced increases in cholesterol accumulation and endolysosome enlargement, we determined next the extent to which cholesterol-enriched diet affected endolysosome function by measuring protein levels and specific activities of three different enzymes; cathepsin D, cathepsin B, and acid phosphatase. We found that the cholesterol-enriched diet increased significantly protein levels of acid phosphatase (**Figure 2A**, *p* < 0.05), cathepsin D (**Figure 2C**, *p* < 0.05), and cathepsin B (**Figure 2E**, *p* < 0.01), but decreased significantly the specific enzyme activity (ratio of enzyme activity to protein levels) of acid phosphatase (**Figure 2B**, *p* < 0.05), cathepsin D (**Figure 2D**, *p* < 0.05), and cathepsin B (**Figure 2F**, *p* < 0.01). Together, these findings indicate that the cholesterol-enriched diet increased the accumulation of cholesterol in endolysosomes and altered the structure and function of endolysosomes in skeletal muscle. As a reminder, we would like to point out that such endolysosome changes are similar to those reported by others in brain neurons from AD patients (Cataldo and Nixon, 1990; Cataldo et al., 1994, 2000) and by us in brains of cholesterol-fed rabbits (Chen et al., 2010).

### Cholesterol-Enriched Diet Increases the Accumulation of Phosphorylated tau, Ubiquitin, and AβPP in Endolysosomes

Multiple proteins that tend to aggregate including phosphorylated tau (Alonso et al., 1996; Alonso Adel et al., 2013; Wang et al., 2013), Aβ (Braak and Del Tredici, 2004; LaFerla et al., 2007), and ubiquitin (Dil Kuazi et al., 2003) are increasingly accumulated in neurons of individuals living with AD. We have shown that these proteins are accumulated in neurons of rabbits fed a cholesterol-enriched diet (Chen et al., 2010). More importantly, we showed previously that these same proteins were also deposited in skeletal muscle of rabbits fed the cholesterol-enriched diet (Chen et al., 2008b). In addition, all these proteins have been linked to endolysosomes; amyloidogenic processing of AβPP has been shown to occur mainly in endolysosomes following AβPP endocytosis (Nixon, 2005; Rajendran and Annaert, 2012; Morel et al., 2013; Jiang et al., 2014), ubiquitin has been shown to function as a signal for membrane protein internalization and protein degradation in the autophagy-lysosome system (Holler and Dikic, 2004; D’Agostino et al., 2011), and tau and phosphorylated tau have been reported to be degraded in the autophagy-lysosome system (Kenessey et al., 1997; Oyama et al., 1998; Hamano et al., 2008; Wang et al., 2009; Chesser et al., 2013). As described above, we demonstrated that cholesterol-fed rabbits markedly enlarged endolysosomes and disturbed their function in skeletal muscle. Thus, we determined next the extent to which cholesterol-enriched diet affected accumulation of these proteins in endolysosomes in skeletal muscle of rabbits fed a cholesterol-enriched diet.

**FIGURE 3:**
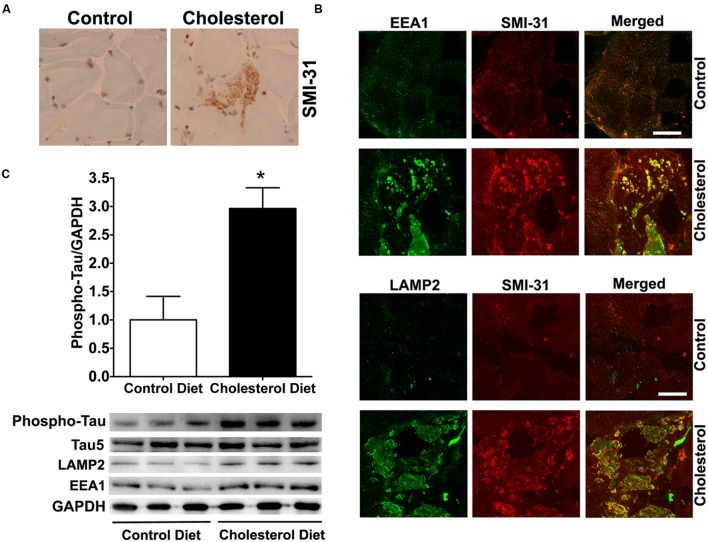
**Cholesterol-enriched diet increases the accumulation of phosphorylated tau in endolysosomes. (A)** In muscle fibers from control rabbits, SMI-31 positive staining was weak, but in muscle fibers from cholesterol-fed rabbits SMI-31 positive staining appeared as large aggregates (40X). **(B)** SMI-31 positive staining of phosphorylated tau (red) co-distributed with EEA1-positive staining of endosomes (green) and LAMP2-positive staining of lysosomes (green) in muscle from cholesterol-fed rabbits. Bar = 20 μm. **(C)** Representative western blots were shown. Rabbits fed cholesterol-enriched diet (*n* = 9) exhibited significantly (^∗^*p* < 0.05) increased protein levels of phosphorylated tau in skeletal muscle, when compared with controls (*n* = 5).

To access endolysosome accumulation of tau protein, we stained phosphorylated tau and found under bright field microscopy that SMI-31 positive phosphorylated tau signals were weak in muscle from control rabbits, but SMI-31 positive phosphorylated tau signals was readily observed and appeared as large aggregates in muscles from cholesterol-fed rabbits (**Figure 3A**). The size and shape of these phosphorylated tau-positive signals were similar to those endolysosome-positive signals (**Figure 1C**), indicating the phosphorylated tau might be accumulating in abnormally enlarged endolysosomes. Using double fluorescence staining methods, we found that intramuscular depositions of phosphorylated tau were co-distributed with EEA1-positive endosomes and LAMP2-positive lysosomes (**Figure 3B**). Our findings are consistent with others’ reports that tau and phosphorylated tau can be degraded in the autophagy-lysosome system (Kenessey et al., 1997; Oyama et al., 1998; Hamano et al., 2008; Wang et al., 2009; Chesser et al., 2013). Although tau and phosphorylated tau are not normally present in early endosomes, under conditions when endocytic homeostasis is disrupted with uncontrolled fusion of early endosomes with late endosomes/lysosomes (**Figure 1D**) it is possible for tau to be co-localized with EEA1 positive early endosomes. These morphological data suggest that cholesterol-enriched diet promotes the intramuscular accumulation of phosphorylated tau in abnormally enlarged endolysosomes. To extend and confirm further our observations, we examined next protein levels of phosphorylated tau using immunoblotting methods. We found significantly increased protein levels of phosphorylated tau (**Figure 3C**, *p* < 0.05) in skeletal muscle from cholesterol-fed rabbits when compared to those from control rabbits. Thus our data suggest that cholesterol-enriched diet leads to an increased accumulation of phosphorylated tau in abnormally enlarged endolysosomes in skeletal muscle.

**FIGURE 4:**
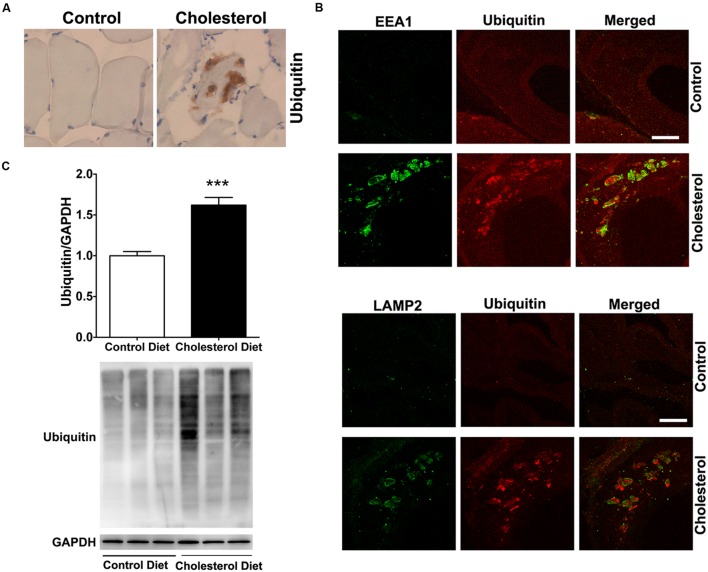
**Cholesterol-enriched diet increases the accumulation of ubiquitin in endolysosomes. (A)** In muscle fibers from control rabbits, ubiquitin positive staining was weak, but in muscle fibers from cholesterol-fed rabbits ubiquitin positive staining appeared as large aggregates (40X). **(B)** Ubiquitin positive staining (red) co-distributed with EEA1-positive staining of endosomes (green) and with LAMP2-positive staining of lysosomes (green) in muscle from cholesterol-fed rabbits. Bar = 20 μm. **(C)** Representative western blots were shown. Rabbits fed cholesterol-enriched diet (*n* = 9) exhibited significantly (^∗∗∗^*P* < 0.001) increased protein levels of ubiquitin in skeletal muscle, when compared with controls (*n* = 5).

To access endolysosome accumulation of ubiquitin, we stained ubiquitin and found under bright field microscopy that ubiquitin positive signals were weak in muscle from control rabbits, but ubiquitin positive staining was readily observed and appeared as large aggregates in muscles from cholesterol-fed rabbits (**Figure 4A**). The size and shape of these ubiquitin positive signals were also similar to those endolysosome-positive signals (**Figure 1C**), indicating the ubiquitin might be accumulating in abnormally enlarged endolysosomes. Using double fluorescence staining methods, we found that intramuscular depositions of ubiquitin were co-distributed with EEA1-positive endosomes and LAMP2-positive lysosomes (**Figure 4B**). These morphological data suggest that ubiquitin accumulates intramuscularly in abnormally enlarged endolysosomes, which are consistent with others’ reports that ubiquitin functions as a signal for membrane protein internalization and protein degradation in the autophagy-lysosome system (Holler and Dikic, 2004; D’Agostino et al., 2011). To extend and confirm further our observations, we examined next protein levels of ubiquitin using immunoblotting methods. We found significantly increased protein levels of ubiquitin (**Figure 4C**, *p* < 0.001) in skeletal muscle from cholesterol-fed rabbits when compared to those from control rabbits. Thus, our data suggest that cholesterol-enriched diet leads to an increased accumulation of ubiquitin in abnormally enlarged endolysosomes in skeletal muscle. The presence of ubiquitin positive inclusions indicate a general degeneration process is occurring. To assess whether such general degeneration might be involved in the pathogenesis of Parkinson’s disease or amyotrophic lateral sclerosis, we determined the expression of α-synuclein and TPD-43 using immunohistochemistry. We found positive but weak immunopositive staining for α-synuclein and TPD-43 in impaired muscle fibers (Supplementary Data). These findings are consistent with our findings that cholesterol-enriched diet induces a general degeneration process as evidenced by the presence of ubiquitin positive inclusions. However, given the weak staining signals for α-synuclein and TPD-43, it is not likely that cholesterol-enriched diet plays a significant or specific pathological role in the development of Parkinson’s disease or amyotrophic lateral sclerosis.

**FIGURE 5:**
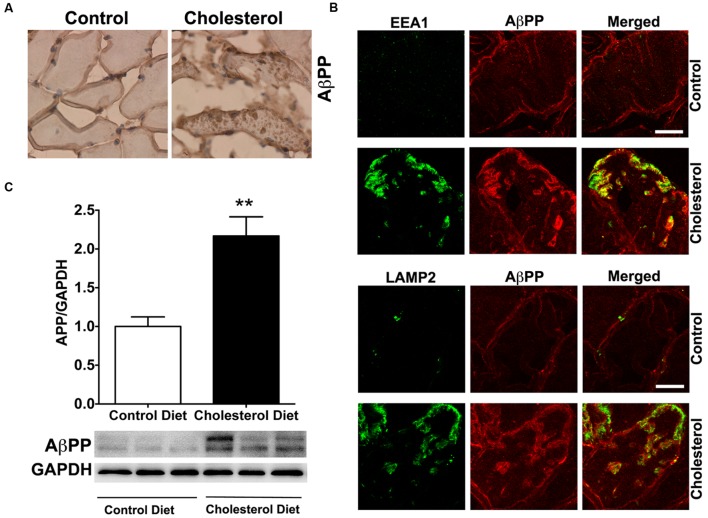
**Cholesterol-enriched diet increases the accumulation of AβPP in endolysosomes. (A)** In muscle fibers from control rabbits, N-terminal AβPP positive signals were mainly present at plasma membrane and intramuscular signals were weak, but in muscles from cholesterol-fed rabbits AβPP positive signals appeared as large aggregates inside muscles (40X). **(B)** N-terminal AβPP positive staining (red) co-distributed with EEA1-positive staining of endosomes (green) and with LAMP2-positive staining of lysosomes (green) in muscle from cholesterol-fed rabbits. Bar = 20 μm. **(C)** Representative western blots were shown. Rabbits fed cholesterol-enriched diet (*n* = 9) exhibited significantly (^∗∗^*P* < 0.01) increased protein levels of AβPP in skeletal muscle, when compared with controls (*n* = 5).

To access endolysosome accumulation of AβPP protein, we stained AβPP with a N-terminal AβPP antibody or a C-terminal AβPP antibody (data not shown) and found under bright field microscopy that AβPP positive signals were mainly present at plasma membrane and intramuscular signals were weak in muscle from control rabbits, but AβPP positive signals were readily observed and appeared as large aggregates inside muscles from cholesterol-fed rabbits (**Figure 5A**). The size and shape of these AβPP positive signals were also similar to those endolysosome-positive signals (**Figure 1C**), indicating the AβPP might be accumulating in abnormally enlarged endolysosomes. Using double fluorescence staining methods, we found that intramuscular depositions of AβPP were co-distributed with EEA1-positive endosomes and LAMP2-positive lysosomes (**Figure 5B**). These morphological data suggest that AβPP accumulates intramuscularly in abnormally enlarged endolysosomes. To extend and confirm further our observations, we examined next protein levels of AβPP using immunoblotting methods. We found significantly increased protein levels of AβPP (**Figure 5C**, *p* < 0.01) in skeletal muscle from cholesterol-fed rabbits when compared to those from control rabbits. Thus, our data suggest that cholesterol-enriched diet leads to an increased accumulation of AβPP in abnormally enlarged endolysosomes in skeletal muscle.

### Aβ Accumulates in Endosomes and Autophagosomes in Skeletal Muscle of Rabbits Fed Cholesterol-Enriched Diet

Endolysosomes play a critical role in amyloidogenic processing of AβPP (Rajendran and Annaert, 2012; Morel et al., 2013; Jiang et al., 2014) and they are major sites where Aβ is generated following internalization of AβPP (Nixon, 2005; Rajendran et al., 2008; Shimizu et al., 2008; Sannerud et al., 2011; Rajendran and Annaert, 2012; Morel et al., 2013; Jiang et al., 2014). Once generated, Aβ can be degraded by cathepsins in lysosomes (Miners et al., 2011) and the remaining Aβ can either accumulate in endolysosomes where it may precipitate AD pathogenesis (Braak and Del Tredici, 2004; LaFerla et al., 2007; Zou et al., 2015) or it can be released via exocytosis (Annunziata et al., 2013; Nilsson et al., 2013). Accordingly, Aβ levels can be enhanced by those factors that promote amyloidogenic processing of AβPP (Grbovic et al., 2003; Ma et al., 2009) and those that impair Aβ degradation (Torres et al., 2012).

**FIGURE 6:**
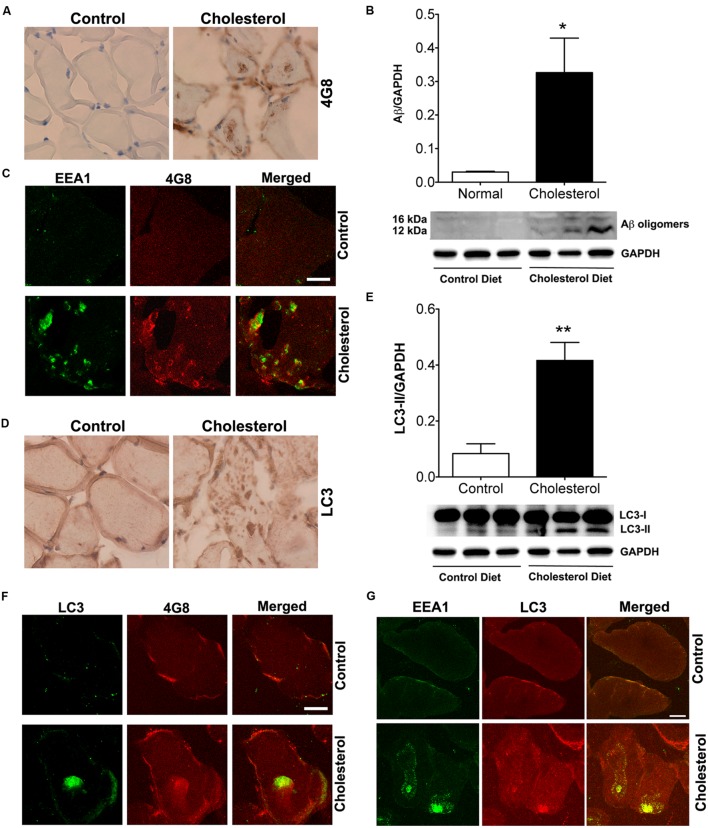
**Cholesterol-enriched diet increases deposition of Aβ in endosomes and autophagosomes. (A)** In muscle fibers from control rabbits, 4G8 positive Aβ signals were weak, but in muscles from cholesterol-fed rabbits 4G8 positive Aβ signals appeared as large aggregates inside muscles (40X). **(B)** Representative western blots were shown. Cholesterol-enriched diet (*n* = 9) increased significantly protein levels of Aβ oligomers (^∗^*p* < 0.05) in skeletal muscle, when compared with controls (*n* = 5). **(C)** 4G8-positive staining of Aβ (red) co-distributed with EEA1 positive staining of endosomes (green) in skeletal muscle from cholesterol-fed rabbits. Bar = 20 μm. **(D)** In muscle fibers from control rabbits, LC3 positive autophagosome signals were weak and diffuse, but appeared as large aggregates inside muscles from cholesterol-fed rabbits (40X). **(E)** Representative western blots were shown. Cholesterol-enriched diet (*n* = 9) increased significantly protein levels of LC3-II (^∗∗^*p* < 0.01) in skeletal muscle, when compared with controls (*n* = 5). **(F)** 4G8-positive staining of Aβ (red) co-distributed with LC3-positive staining for autophagosomes (green) in skeletal muscle from cholesterol-fed rabbits. Bar = 20 μm. **(G)** EEA1positive endosomes (green) co-distributed with LC3-positive staining for autophagosomes (red) in skeletal muscle from cholesterol-fed rabbits. Bar = 20 μm.

Given our findings that cholesterol enriched diet increases cholesterol accumulation in endolysosomes and dramatically altered structure and function of endolysosomes, we determined the extent to which cholesterol-enriched diet affects levels of intramuscular Aβ. Using the 4G8 antibody for detection of Aβ, we found under bright field microscopy that 4G8 positive Aβ signals appeared as large aggregates inside muscles from cholesterol-fed rabbits (**Figure 6A**). Using immunoblotting methods, we found that cholesterol-enriched diet increased significantly protein levels of Aβ oligomers (**Figure 6B**, *p* < 0.05) in skeletal muscle from cholesterol-fed rabbits. Using double fluorescent staining, we found that 4G8-positive Aβ staining co-distributed with EEA1-positive endosomes (**Figure 6C**), a finding consistent with endosome production of Aβ. None of these features was present in skeletal muscle of control rabbits. Our observations of increased accumulation of Aβ/AβPP in endolysosomes suggests to us that increased amyloidogenic processing of AβPP was caused by morphological and functional alterations in endolysosomes.

Because Aβ was also found to be a substrate for autophagy (Nixon, 2007), we next determined the extent to which Aβ accumulated in autophagosomes. We found under light microscopy that LC3 positive autophagosome signals were weak and diffuse in muscles from control rabbits, but appeared as large aggregates inside muscles from cholesterol-fed rabbits (**Figure 6D**). Using immunoblotting methods, we found that cholesterol-enriched diet increased significantly protein levels of LC3-II, an indicator of autophagosome proliferation and maturation (**Figure 6E**, *p* < 0.01). Using double fluorescent staining, we found that 4G8-positive Aβ staining co-distributed with LC3 positive autophagosomes (**Figure 6F**). The observation that Aβ is present in LC3-positive autophagosomes suggests further the presence of dysfunctional endolysosome-autophagosome system. Given our finding that cholesterol-enriched diet increases the co-localization of EEA1 with LAMP2 (**Figure 1D**), which indicates endolysosome accumulation of cholesterol could lead to uncontrolled fusion of early endosomes with late endosome/lysosomes, we determined the extent to which cholesterol-enriched diet affects the co-localization of EEA1 with LC3. We found that cholesterol-enriched diet increased dramatically co-localization of EEA1 with LC3 (**Figure 6G**). These observations indicate that endolysosome accumulation of cholesterol could also increase fusion of early endosomes with autophagosome, a phenomenon that fosters maturation of autophagosomes (Tooze and Razi, 2009).

## Discussion

The present studies were aimed to test the hypothesis that endolysosome dysfunction in skeletal muscle shares common pathological features to those found in brain in a rabbit model of sporadic AD. Principally, we demonstrated that skeletal muscle from a cholesterol-fed rabbit model of AD exhibited increased cholesterol accumulation in endolysosomes, enlarged endolysosomes, and impaired endolysosome function. Furthermore, we demonstrated that various AD marker proteins including phosphorylated tau, ubiquitin, AβPP and Aβ were accumulated in those enlarged endolysosomes. Together these findings provide further insight into skeletal muscle involvement in AD and the potentially significant role that endolysosomes play in the pathogenesis of AD.

Endolysosomes are acidic organelles consisting of endosomes, lysosomes, and autophagosomes that play a key role in protein turnover and cellular homeostasis (Appelqvist et al., 2013). Substrates for degradation are delivered to lysosomes by two general routes namely endocytosis and autophagy. Endocytosis is responsible for up-taking extracellular nutrients as well as turnover of plasma membrane proteins. Autophagy, on the other hand, is responsible for removing intracellular protein aggregates and “worn out” organelles. Endolysosomes are especially important for physiological functions of neurons and skeletal muscle cells because they are long-lived post-mitotic cells that require efficient endolysosomes to degrade intracellular protein aggregates, eliminate “worn out” organelles and maintain membrane integrity. As such, endolysosome dysfunction in neurons has been linked to neurodegeneration and has been shown to play an early and important role in the development of AD (Cataldo et al., 2000; Nixon, 2005; Rajendran et al., 2008; Shimizu et al., 2008; Sannerud et al., 2011). Similarly, endolysosome dysfunction in skeletal muscle contributes to muscle degeneration and age-related disorders and has been implicated in a variety of autophagosome and lysosome related myopathies (Askanas et al., 2009; Nogalska et al., 2010; Malicdan and Nishino, 2012; Bonaldo and Sandri, 2013; Demontis et al., 2013; Sandri et al., 2013). Interestingly, skeletal muscle deficits such as loss of muscle mass and reduced muscle strength has been shown to be early signs of AD that contribute to disability and could predict the onset and progression of clinical AD (Buchman et al., 2007; Boyle et al., 2009; Burns et al., 2010). However, the question of whether endolysosome dysfunction leads to development of pathological features of AD in skeletal muscle and contributes to skeletal muscle deficits in AD has not been addressed. As such, we determined the extent to which elevated levels of plasma LDL cholesterol, a robust risk factor for AD, disturbed the structure and function of endolysosomes and promoted the development of AD-like pathological features including intracellular deposition of Aβ/AβPP, phosphorylated tau, and ubiquitin in skeletal muscle.

Given that plasma lipoproteins supply skeletal muscle with needed cholesterol (Spady and Dietschy, 1983), elevated plasma LDL cholesterol and subsequent increases in LDL cholesterol uptake could lead to increased cholesterol accumulation in endolysosomes and disturbed endolysosome structure and dysfunction. As expected, we demonstrated cholesterol-enriched diet increased free cholesterol in endosomes and lysosomes in skeletal muscle. Importantly, we demonstrated that cholesterol-enriched diet dramatically enlarged endolysosomes and inhibited endolysosome function. Our observations that cholesterol-enriched diet increased co-localization of EEA1 with LAMP2 and LC3 indicate that abnormally enlarged endolysosomes might result from uncontrolled fusion of early endosomes with late endosome/lysosomes or autophagosomes, a finding that is consistent with recent reports that EEA1 positive early endosomes could fuse with lysosomes under disrupted endocytic homeostasis (Falcon-Perez et al., 2005; Das and Pellett, 2011; Ramanathan et al., 2013).

The observations that cholesterol enriched diet increased accumulation of Aβ/AβPP in endolysosomes suggest to us that increased amyloidogenic processing of AβPP was caused by morphological and functional alterations in endolysosomes. The observation that Aβ is also present in LC3-positive autophagosomes suggests further the presence of dysfunctional endolysosomes because autophagic activity itself is part of the endolysosome system. Elevated levels of LDL cholesterol could promote Aβ deposition in skeletal muscle in two ways. First, receptor-mediated endocytosis of LDL cholesterol could encourage the accumulation of AβPP in endolysosomes by either promoting AβPP internalization (Waldron et al., 2006, 2008; Klug et al., 2011) or impairing the recycling of internalized AβPP back to plasma membrane (Andersen et al., 2005; Willnow and Andersen, 2013). Second, endolysosome inhibition could lead to impairment of Aβ degradation in lysosomes because Aβ can be degraded in lysosomes by cathepsin D and B (Nogalska et al., 2012; Saido and Leissring, 2012). Our observations that cholesterol-enriched diet increases endolysosome accumulation of AβPP and decreased specific enzyme activities of cathepsin D and B support, at least indirectly, both possible mechanisms.

The observations that cholesterol-enriched diet increased accumulation of ubiquitin and phosphorylated tau in endolysosomes in skeletal muscle indicate that endolysosome dysfunction as induced by a cholesterol-enriched diet contributes to the development of AD-like ubiquitin-positive multi-protein aggregates and inclusions in skeletal muscle. These findings are consistent with other’s reports that increased accumulation of cholesterol in lysosomes and subsequent lysosome dysfunction has been linked to the development of neurofibrillary tangles in brains of patients with Niemann-Pick type C disease (Sawamura et al., 2001; Bu et al., 2002; Distl et al., 2003; Vance, 2006; Bi and Liao, 2007; Liao et al., 2007) and our reports that endolysosome dysfunction as induced by high levels of LDL cholesterol contributes to the development of tau-pathology in neurons (Chen et al., 2010; Hui et al., 2012).

Although a causal relationship is not determined, our findings do suggest that endolysosome dysfunction as induced by elevated plasma LDL cholesterol, aging, or other factors may play a pathogenic role in the development of AD-like pathological changes observed in skeletal muscle, similar to its role in neurodegeneration and the pathogenesis of AD in brain. Furthermore, our findings support the notion that common pathogenic mechanisms may exist in skeletal muscle and neurons, and that the skeletal muscle changes may represent early and progressive pathological features of AD.

## Author Contributions

XC, OG, and JG contributed to design the work. XC and JW contributed to the acquisition and analysis of data. XC drafted the work. XC, JW, OG, and JG contributed to interpretation of data and revising the work of intellectual content and final approval of the version to be published. XC drafted the work. XC, JW, OG, and JG approved of the final version and agreed to be accountable for all aspects of the work in ensuring that questions related to the accuracy or integrity of any part of the work are appropriately investigated and resolved.

## Conflict of Interest Statement

The authors declare that the research was conducted in the absence of any commercial or financial relationships that could be construed as a potential conflict of interest.
