# Supplementary material for: Role of Endolysosomes in Skeletal Muscle Pathology Observed in a Cholesterol-Fed Rabbit Model of Alzheimer’s Disease
Source: Front Aging Neurosci. 2016 Jun 8;8:129. doi: 10.3389/fnagi.2016.00129 (PMC4896918; doi:10.3389/fnagi.2016.00129)
Supplement: Supplementary file 1 [file Image_1.PDF]

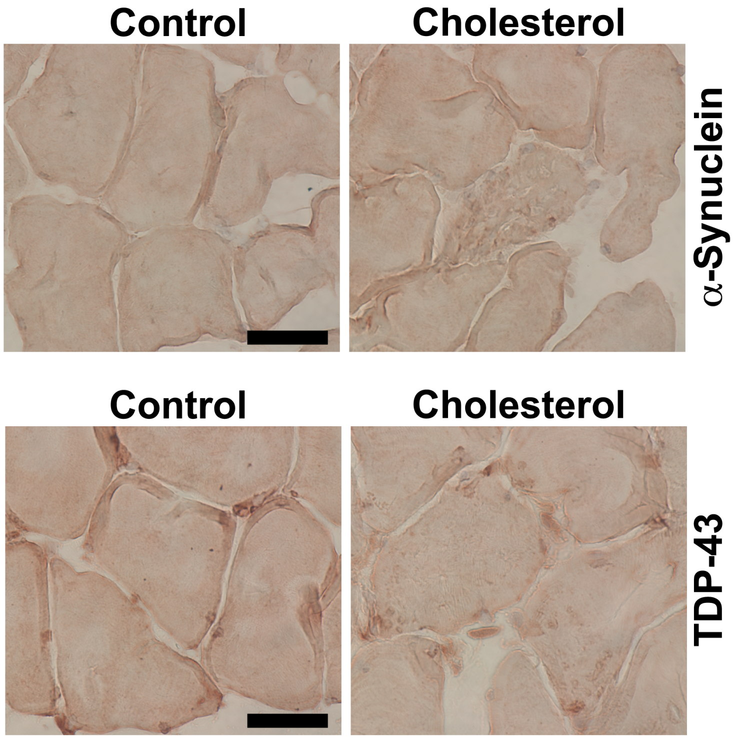

Supplementary data: The expression of α-synuclein and TDP-43 was determined using immunohistochemistry with mouse anti-alpha synuclein (Abcam, ab27766) and mouse anti-TDP43 (Abcam, ab57105) antibodies respectively. Positive but weak immunopositive staining for α-synuclein and TDP-43 in impaired muscle fibers from cholesterol-fed rabbits were observed (40X).
